# Echolocating Big Brown Bats, *Eptesicus fuscus*, Modulate Pulse Intervals to Overcome Range Ambiguity in Cluttered Surroundings

**DOI:** 10.3389/fnbeh.2016.00125

**Published:** 2016-06-22

**Authors:** Alyssa R. Wheeler, Kara A. Fulton, Jason E. Gaudette, Ryan A. Simmons, Ikuo Matsuo, James A. Simmons

**Affiliations:** ^1^Department of Neuroscience, Brown UniversityProvidence, RI, USA; ^2^Circuit Dynamics and Connectivity Unit, National Institute of Neurological Disorders and Stroke, National Institutes of HealthBethesda, MD, USA; ^3^Sensors and Sonar Systems Department, Naval Undersea Warfare CenterNewport, RI, USA; ^4^Department of Biostatistics and Bioinformatics, Duke University School of MedicineDurham, NC, USA; ^5^Department of Information Science, Tohoku Gakuin UniversitySendai, Japan

**Keywords:** echolocation, sonar, clutter, bat, range ambiguity

## Abstract

Big brown bats (*Eptesicus fuscus*) emit trains of brief, wideband frequency-modulated (FM) echolocation sounds and use echoes of these sounds to orient, find insects, and guide flight through vegetation. They are observed to emit sounds that alternate between short and long inter-pulse intervals (IPIs), forming sonar sound groups. The occurrence of these strobe groups has been linked to flight in cluttered acoustic environments, but how exactly bats use sonar sound groups to orient and navigate is still a mystery. Here, the production of sound groups during clutter navigation was examined. Controlled flight experiments were conducted where the proximity of the nearest obstacles was systematically decreased while the extended scene was kept constant. Four bats flew along a corridor of varying widths (100, 70, and 40 cm) bounded by rows of vertically hanging plastic chains while in-flight echolocation calls were recorded. Bats shortened their IPIs for more rapid spatial sampling and also grouped their sounds more tightly when flying in narrower corridors. Bats emitted echolocation calls with progressively shorter IPIs over the course of a flight, and began their flights by emitting shorter starting IPI calls when clutter was denser. The percentage of sound groups containing 3 or more calls increased with increasing clutter proximity. Moreover, IPI sequences having internal structure become more pronounced when corridor width narrows. A novel metric for analyzing the temporal organization of sound sequences was developed, and the results indicate that the time interval between echolocation calls depends heavily on the preceding time interval. The occurrence of specific IPI patterns were dependent upon clutter, which suggests that sonar sound grouping may be an adaptive strategy for coping with pulse-echo ambiguity in cluttered surroundings.

## Introduction

Big bats (*Eptesicus fuscus)* emit sequences of brief, wideband frequency-modulated (FM) ultrasonic calls and use echoes of these sounds to orient, find insects, and guide flight through the surrounding environment. The resulting sequences of echoes yield successive views of the scene as it unfolds before the bat in flight. Faster rates of echolocation sound production result in more frequent updates of the scene. Bats actively adjust their sonar broadcasts to compensate for a multitude of environmental challenges, including the movement of targets, the presence of obstacles, and reception of echoes from vegetation and other background objects, called *clutter* (Denny, 2007; Petrites et al., 2009; Moss et al., 2011; Kothari et al., 2014). The distance to the farthest detectable objects, the proximity of the nearest objects, and the density of objects comprising the scene, as well as other factors, affect the echolocation call parameters needed to perceive a complex and dynamic environment (Surlykke and Moss, 2000; Moss and Surlykke, 2001; Petrites et al., 2009; Simmons, 2014).

Big brown bats detect insects from about 5 m away, and track them until they are 10 cm away or less before the moment of capture (Kick and Simmons, 1984). However, larger objects and surfaces such as background vegetation or the ground are detectable at ranges up to 30–40 m (Stilz and Schnitzler, 2012). A bat flying in pursuit of an insect emits echolocation sounds rapidly enough to track the moving target on the approach, but if the bat receives echoes from objects located far away, it may need to wait for those echoes to return before emitting the next sound. To image the entire acoustic scene and update the image rapidly enough to accommodate changing conditions, the bat has to resolve these two competing requirements: the bat must emit sounds slowly enough to detect echoes from distant objects, but it must emit sounds rapidly enough to track nearby moving objects. A radar or sonar system generally balances these two requirements by emitting a mixture of sound pulses with long intervals between them (inter-pulse intervals) and other sounds with short intervals between them (Stimson, 1998; Denny, 2007). This way, long-range echoes from distant objects arrive during a long inter-pulse interval (IPI), and echoes from short-range or moving objects arrive during a short IPI. However, this strategy is subject to disadvantages when the scene contains numerous, densely packed objects (clutter) at a wide variety of distances.

In situations where multiple echoes arrive from clutter, big brown bats broadcast their sounds more frequently to better discriminate prey echoes and avoid collisions (Petrites et al., 2009; Hiryu et al., 2010; Moss and Surlykke, 2010; Falk et al., 2011, 2014; Moss et al., 2011; Kothari et al., 2014; Sändig et al., 2014). They also emit groups of sounds (*strobe groups* or *sonar sound groups*), mostly in pairs or triplets, that are defined by short, stable within-group IPIs and are separated from one another by long IPIs (Surlykke and Moss, 2000; Moss and Surlykke, 2001; Moss et al., 2006; Saillant et al., 2007; Petrites et al., 2009; Hiryu et al., 2010). These bats commonly are observed to emit strobe groups when tracking a target—especially amongst clutter (Moss et al., 2006; Kloepper et al., 2014; Kothari et al., 2014; Sändig et al., 2014). Moreover, when the surrounding scene contains obstacles in close proximity while the scene as a whole has obstacles distributed over a wide span of distances, strobe groups dominate the stream of echolocation sounds (Petrites et al., 2009). This evidence suggests that sonar sound grouping behavior may be related to the conflict between perceiving the whole scene and avoiding close-proximity obstacles. However, emitting sounds in strobe groups results in the bat not waiting for all echoes return from one call (or “pulse”) before emitting the next pulse. Overlap of the echo-reception epochs for two successive sounds creates *pulse-echo ambiguity* (also called *range ambiguity*, or *pulse-echo overlap*; Stimson, 1998; Denny, 2007) about which of the echolocation broadcasts actually produced which echoes. This is a serious problem for sonar and airborne radar systems and has also been considered a potential problem for echolocation (Kalko and Schnitzler, 1998; Stimson, 1998; Denny, 2007; Petrites et al., 2009; Hiryu et al., 2010; Melcón et al., 2011). Echolocation pulse grouping in a cluttered scene seems to be an adaptive strategy for echolocating in surroundings that can produce ambiguous echoes as a consequence of overlap between echo-receiving epochs for successive pulses. The use of so many pulses in a short timespan would create sporadic ambiguous echoes, but the absence of those ambiguous echoes from most of the echo streams may allow the bat to ignore them. By emitting signals in a pattern of long and short IPIs in succession (sonar sound groups), bats alternate between sampling the long-range and the short-range environment.

The experiment described here was carried out to measure the patterning of sonar sound groups during flight in very dense, extended clutter. Big brown bats emit occasional strobe groups when the surrounding clutter is more than 1 m away, but then abruptly shift to emitting the majority of their sounds in groups when clutter is 1 m or less away (Petrites et al., 2009). This shift suggests that a new mode of echolocation behavior is triggered in close-proximity clutter, and this experiment was designed to examine how this behavior changes as obstacles are extremely close—so close as to barely accommodate the bat's wingspan. The current study was designed without an insect target in order to examine obstacle-avoidance, navigation, and path-planning orientation behavior. We used rows of hanging plastic chains that filled a large volume of a flight room and produced vegetation-like acoustic reflections. This type of dense object array has been shown to be ideal for maximizing sonar sound grouping behavior (Petrites et al., 2009). The effects of clutter on bat echolocation behavior were investigated using this obstacle matrix, and were measured by the inter-pulse intervals emitted during flight through the array. We hypothesized that a decreased distance between the bat and surrounding objects would decrease IPIs, and increase sonar sound group prevalence and size.

## Methods

### Animal subjects

Four adult male big brown bats (*Eptesicus fuscus*) naïve to the procedure were flown in this study. These bats were wild-caught in Rhode Island under scientific collecting permits #2012-34 and #2013-32 issued by the state Department of Environmental Management. Bats were housed in a temperature and humidity-controlled colony room (22–24°C, and 60–75% relative humidity), which was set to a 12 h/12 h reversed dark/light cycle in order to perform experiments during the day on alert bats. Bats were maintained individually on a diet of mealworms, larval *Tenebrio molitor*, which were adjusted for each bat in order to maintain a mass between 15 and 18 g. All bats had free access to vitamin-supplemented water (Poly-vi-sol) refreshed daily. The Brown University Institutional Animal Care and Use Committee approved all experimental procedures and animal care protocols.

### Flight room

**Figure 1:**
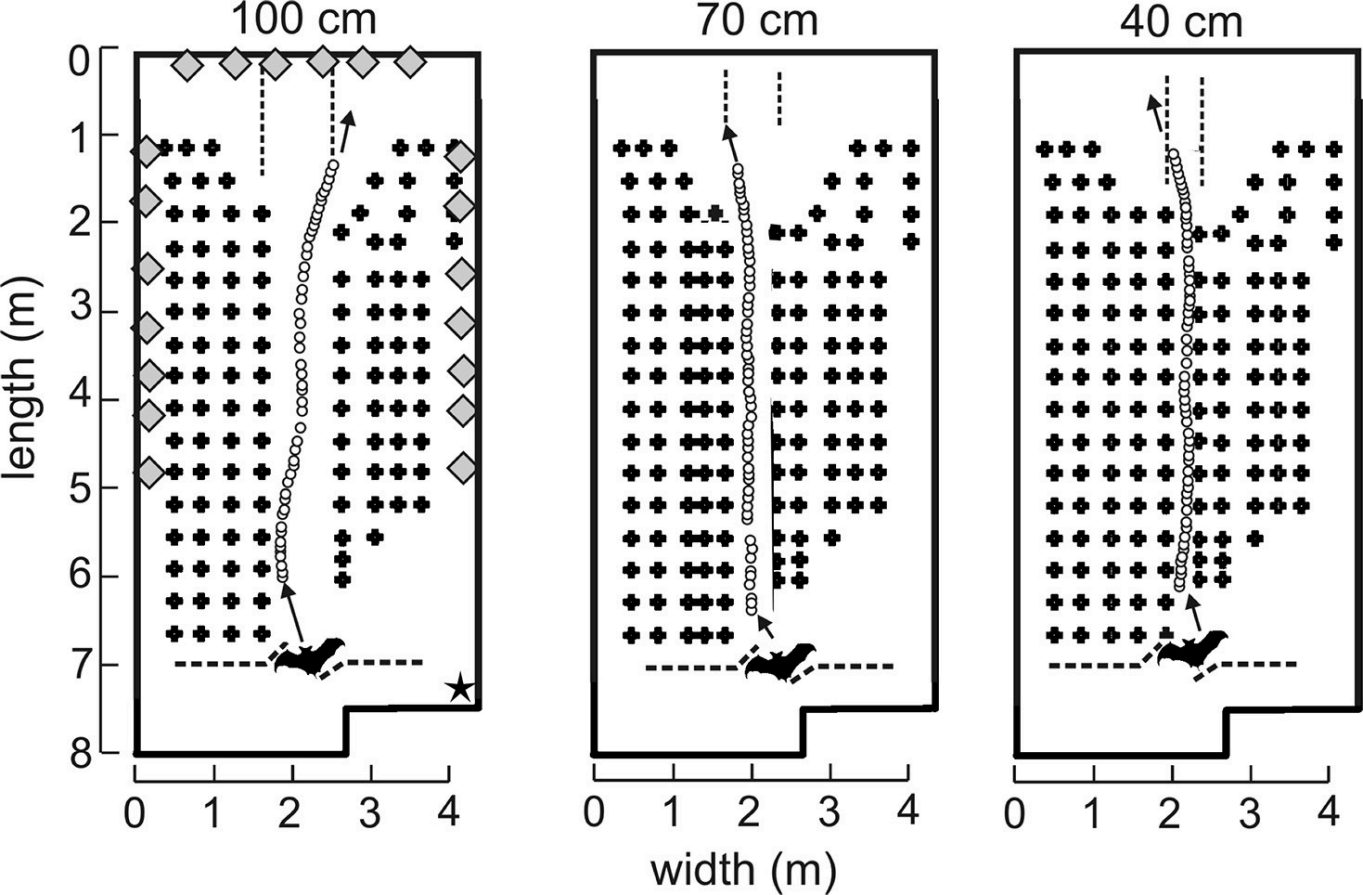
**Experimental flight room diagrams and example flights**. Plan view of flight room (8.3 m long, 4.3 m wide, and 2.7 m high). Rows of vertically hanging plastic chains (plus signs, not to scale) were spaced 40 cm apart. Each bat was released through an opening in the net (dashed line at bottom) and flew down the corridor (spaced apart by 100, 70, or 40 cm) in the direction of the arrow to land on the back wall. Pulse emission locations (open circles) from an example bat (not to scale, bats wingspan = 30–32 cm) in one representative flight from each corridor were determined by time-difference of arrival (TDOA) measurements from 20 microphones distributed around the room (gray diamonds, not to scale). Microphones appearing next to one another in this diagram were spaced apart vertically. An infrared camera (star) was located in the top right corner of the front of the room such that bat flights could be monitored in real time.

All flight experiments were performed in a custom-built flight room insulated acoustically and electrically from outside noise. The flight room measured 8.3 m long by 4.3 m wide by 2.7 m high (Figure 1). A vertical net was suspended from the ceiling about 7 m from the far wall to separate the flight volume from the experimenter who released the bat on each flight. The net was cut in the center to allow the experimenter to reach forward and release the bat into the active flight volume (Figure 1). A 30 frame-per-second infrared camera (Photon 320, FLIR, Billerica, MA) with a 19-mm lens affording a 36° field of view was placed in the upper right-front corner of the room to record the bats flight so that experimenters in the adjacent electronic control room could observe in real time while turning the recording system on and off for each flight. All walls and the ceiling were entirely covered in light gray fireproof anechoic acoustic foam (Sonex “One” panels, West General LLC, San Jose, CA) to absorb 20–25 dB of the sound energy from incident echolocation sounds and prevent strong wall and ceiling echoes from reaching the bat. Multiple rows of black plastic chains were suspended vertically from foam-covered metal crossbars near the ceiling of the flight volume to act as vegetation-like acoustic reflectors for clutter (Petrites et al., 2009). The individual oval chain links measured 4.0 cm wide, 7.5 cm long, and 1.0 cm thick. Left-to-right, the chains in each row were 30 cm apart, and along the flight path (front-to-back) the successive rows were separated by 40 cm. The big brown bat's maximum wingspan is about 30–32 cm. The spacing of the chains was chosen to deter the bat from turning part-way along the obstacle-free corridor to fly left or right between rows and instead keep to flying along the corridor to the far end of the room. The configuration of these chains in multiple rows for each experimental condition is illustrated in Figure 1. The width of the corridor was manipulated while maintaining even chain spacing by adding or removing columns of chains in the center of the array. As the width of the corridor was narrowed (100, 70, and 40 cm), the nearest clutter to the flying bats became closer. Moreover, because the rows of chains extend down the room for 5 meters, the collision hazard was present throughout the majority of each flight.

### Sound recording

Twenty electret microphones picked up the sonar sounds emitted by the bat during flight with good ultrasonic sensitivity (MEMS SPM0404UD5, Knowles Electronics, Itasca, IL). These were individually mounted on custom-built preamplifier and high-pass filter boards that were distributed around the left and right side walls and the far wall of the flight room (diamonds, Figure 1). Each microphone-preamplifier unit was fitted with a 20-cm square foam baffle to minimize backscatter from the wall located behind each microphone. Outputs of all 20 channels were digitized using a commercial audio processor (one PCIe-424 with two accompanying HD192, all from MOTU, Cambridge, MA) at a sampling rate of 192 kHz and stored as.wav files on a devoted computer (Mac Pro Early 2009/MacPro4.1, Apple, Cupertino, CA). All electronic wiring from microphones and the infrared camera were fed through a small opening to an adjacent room where acoustic and visual signals were displayed and recorded. Experimenters in the adjacent control room began the sound recording when instructed by the experimenters in the flight room, and stopped recording when the bat landed on the far wall as visualized in real time by the infrared camera.

### Experimental procedure

The experiment was conducted between September 20th and November 27th, 2013. Before starting the experiment, bats were trained to fly from a release point at the front of the room (Figure 1) and traverse the room to land on the back wall. Bats were rewarded with a mealworm for each successful flight, with a maximum of 10 successful flights per day. A trial was considered to be a successful flight when the bat navigated the obstacle matrix in its entirety without leaving the corridor and without colliding with chains. This includes flights where the bat navigated the chain matrix, turned around at the back wall without landing, and re-entered the array. For these “looping” trials, only complete forward (in the original direction of release) navigations of the chain array were included in the analysis. Thus, each bat was released for a maximum of 10 successful trials per day, but looping behavior during a successful trial allowed for more than 10 “flights” eligible for data analysis. All flights were performed in the dark to exclude visual cues from the bats' perception. The flight room was completely dark with the exception of a single dim long-wavelength light (>650 nm) in the back left corner of the room near the back wall landing site. The *E. fuscus* retina is insensitive to this type of light (Hope and Bhatnagar, 1979), yet it allowed experimenters to see and retrieve the bat after each trial. The corridor was progressively narrowed over time from 100 to 70 cm, and finally 40 cm during Block 1. The narrowest corridor width was 40 cm as this was the narrowest condition safely navigable by the bats. We then repeated this procedure in reverse going from 40 to 70 cm to 100 cm during Block 2.

### Pulse-interval analysis

Sounds recorded by the single microphone on the end (i.e., landing) wall in the room array that best exhibited consistency of the signal amplitude throughout the trial were analyzed for inter-pulse intervals (IPIs). Only successful flights as defined in 2.4 above were analyzed. All sound files were truncated where the bat made a terminal “landing” buzz as visualized by a steady decrease in pulse amplitude and simultaneous decrease in IPI. In cases where the bat looped back along the corridor from the original release point, a new flight was delineated. A custom program (MATLAB, MathWorks, Cambridge, MA) identified all sounds, and then used a low-pass frequency filter below 35 kHz and a high-pass filter above 50 kHz to separate communication sounds from echolocation calls, respectively. The envelope of each sound then was computed using the Hilbert transform. Moving averages from the envelope were used to smooth the low-passed pattern, and individual echolocation sounds were detected by using a threshold higher than the background of low-passed noise. The program then extracted IPIs from the time between peaks for each sound, and IPIs were filtered to include only those between 12 and 100 ms. This was done to prevent the MATLAB program from erroneously registering strong echoes as calls, which would create very short IPIs, and to exclude sounds made while still in the experimenter's hand, or after landing, which typically have IPIs longer than 100 ms. Figure 1 shows example pulses made by one example bat (Bat 4) for one flight in each condition, as measured by time difference of arrival (TDOA).

### Statistical analyses

After measuring IPIs, statistical modeling and figures were generated using SAS/STAT® or R (R Core Team, 2014) statistical packages. Due to the hierarchical nature of the inter-pulse interval dataset (bat, day, flight, and call within a flight) we aimed to fit a generalized linear mixed model (GLMM). However, since the IPIs were truncated, or filtered to exclude IPIs less than 12 ms and greater than 100 ms, it was necessary to first investigate the potential for bias in such a model, because a GLMM makes parametric assumptions. To examine the potential for bias, both a generalized linear model (GLM) and a truncated regression model (TRM; Amemiya, 1973) were fit to the IPI dataset in SAS using the following parameters:

$$\begin{array}{l} \eta =\mu +\beta_{1}Call_{i}+\beta_{2}I\left(CW=40\right)+\beta_{3}I\left(CW=70\right)+\epsilon_{i} \end{array}$$

where η = E[Y_*i*_] is the expected pre-IPI (IPI) of call *I* and ϵ_*i*_ is the error term. A substantial degree of bias was found for the GLM (see Table S1), but deemed acceptable since the direction of bias underestimates, rather than overestimates, the effects of call number and corridor width. Therefore, use of a GLM underestimates the effect size and reduces the variance of any associations, while providing a framework for the inclusion of random effects in a generalized linear mixed model (GLMM), which would otherwise not be possible using a TRM. An interaction between call number and corridor width was discovered (see Figure S3), whereby the slope of pre-IPI over successive calls within a flight was different in each corridor width. This interaction term was included in a generalized linear mixed model (GLMM) that was fit to describe the effect of corridor width on IPI.

#### Generalized linear mixed model of IPI and IPI ratio

In the GLMM, bat, day, and flight are treated as nested random effects. We parameterize this model as:

$$\begin{array}{l} \eta =\mu +\beta_{1jkl}Call_{ijkl}+\beta_{2}I\left(CW=40\right)+\beta_{3}I\text{​}\left(CW=70\right)\\ \;+\text{​}\beta_{1jkl}\beta_{2}Call_{ijkl}I\left(CW=40\right)+\beta_{1jkl}\beta_{3}Call_{ijkl}I\text{​}\left(CW\text{​}=\text{​}70\right)\\ \;+\alpha_{l}+\gamma_{kl}+\delta_{jkl}+\epsilon_{ijkl} \end{array}$$

where η = E[Y_*ijkl*_] is the expected pre-IPI (IPI) of call *i* on flight *j*, on day *k* for bat *l*, and μ represents the average pre-call IPI (pre-IPI) or across all levels (i.e., model intercept). β_1*jkl*_ is the slope pre-IPI across calls within flight *j*, day *k*, and bat *l. I(CW* = *X*) is a binary indicator variable for *X* cm corridor width. β_2_ represents the additive effect of 40 cm corridor width compared to 100 cm corridor width, while β_3_ represents the similar effect for 70 cm corridor width. Interaction terms between call and corridor width are modeled by β_1**jkl**_ β_2_ and β_1**jkl**_ β_3_. The random effects are α_*l*_, γ_*kl*_, and δ_*jkl*_, and ϵ_*ijkl*_ is the error term. Random intercepts are α_*l*_ ~ *N*(0, ψ) for bat *l*, γ_*kl*_ ~ *N*(0, ϕ) for day *k* for bat *l*, and δ_*jkl*_ ~ *N*(0, ξ) for flight *j*, on day *k*, for bat *l*.

A generalized linear mixed model (GLMM) was also fit to investigate the hierarchical structure of the IPI ratio (post-IPI/pre IPI) for each call with respect to individual bat and corridor width. In this model, bat, day, and flight are treated as nested random effects. We parameterize this model as:

$$\begin{array}{l} \eta =\mu +\beta_{1jkl}Call_{ijkl}+\beta_{2}I\left(CW=40\right)+\beta_{3}I\left(CW\text{​}=\text{​}70\right)\\ \;+\text{​​}\beta_{1jkl}\beta_{2}Call_{ijkl}\;I\left(CW\text{ ​}=\text{​ }40\right)+\beta_{1jkl}\beta_{3}Call_{ijkl}\;I\left(CW\text{​}=\text{​}70\right)\\ \;+\alpha_{l}+\gamma_{kl}+\delta_{jkl}+\epsilon_{ijkl} \end{array}$$

where η = E[Y_*ijkl*_] is the expected IPI ratio of call *i*, on flight *j*, on day *k*, for bat *l*, and μ represents the average IPI ratio across all levels (i.e., model intercept). β_1*jkl*_ is the slope of IPI ratio across calls within flight *j*, day *k*, and bat *l. I(CW* = *X*) is a binary indicator variable for *X* cm corridor width. β_2_ represents the additive effect of 40 cm corridor width compared to 100 cm corridor width, while β_3_ represents the similar effect for 70cm corridor width. Interaction terms between call and corridor width are modeled by β_1**jkl**_ β_2_ and β_1**jkl**_ β_3_. The random effects are α_*l*_, γ_*kl*_, and δ_*jkl*_, and ϵ_*ijkl*_ is the error term. Random intercepts are α_*l*_ ~ *N*(0, ψ) for bat *l*, γ_*kl*_ ~ *N*(0, ϕ) for day *k* for bat *l*, and δ_*jkl*_ ~ *N*(0, ξ) for flight *j*, on day *k*, for bat *l*.

#### Finite mixture model of IPI ratio distribution

While the GLMM was used because it allowed the hierarchical correlation structure of the data to be included in the model (bat, day, etc.), **Figure 5B** shows that the distribution of IPI ratios is approximately lognormal. A log transform of these IPI ratio data revealed bimodality (**Figure 5C**). To investigate the effects of corridor width on the IPI ratio distribution, a finite mixture model (FMM) was fit to the log-transformed IPI ratio data. This approach modeled log (IPI ratio) as a combination of two separate normal distributions, implying a latent or hidden process that modulated whether the ratio was part of one distribution or the other. This model explicitly assumed that these log-transformed IPI ratio values were distributed according to a mixture of two normal distributions. The FMM was able to provide a more detailed analysis of the IPI ratio data than could be offered by the GLMM.

#### Strobe group analysis

Sonar sound groups were identified and defined by criteria developed by Kothari et al. (2014). In this method, sonar sound groups have a 5% tolerance for within-group IPIs (“stability criterion”—only applicable to groups with three or more sounds), and must be separated by an IPI of at least 1.2 times the mean within-group IPI (“island criterion”). Calls were categorized as singles or as belonging to a sonar sound group using a custom MATLAB program. In this analysis, the maximum sonar sound group size was a quadruplet (4 sounds per group). Proportions of sounds belonging to singles, doublets, triplets, or quadruplets were averaged across bats for each corridor width and subjected to chi-square analysis.

## Results

A total of 44,998 IPIs were analyzed from sound recordings made during 765 bat flights in the three experimental conditions. Bat 1 successfully completed 185 of 189 total flights (23, 79, and 83 in the 100, 70, and 40 cm corridors respectively). Bat 1 emitted an average of 28.4 calls/s in the 100 cm corridor, 26.1 calls/s in the 70 cm corridor, and 26.1 calls/s in the 40 cm corridor. Bat 1 flew at 3.8, 3.6, and 3.8 m/s on average in the 100, 70, and 40 cm corridors, respectively.

Bat 2 completed 186 of 192 flights (50, 73, and 63 flights in the 100, 70, and 40 cm corridor widths, respectively. Bat 2 emitted an average of 32.0 calls/s in the 100 cm corridor, 35.4 calls/s in the 70 cm corridor, and 40.5 calls/s in the 40 cm corridor. Bat 2 flew at 3.6, 4.1, and 3.7 m/s on average in the 100, 70, and 40 cm corridors, respectively. Bat 3 completed 159 of 162 flights (41, 62, and 56 flights in the 100, 70, and 40 cm corridors, respectively).

Bat 3 emitted an average of 25.9 calls/s in the 100 cm corridor, 27.4 calls/s 70 cm corridor, and 31.7 calls/s in the 40 cm corridor. Bat 3 flew at 4.1, 4.0, and 3.4 m/s on average in the 100, 70, and 40 cm corridors, respectively.

Bat 4 completed 235 of 240 flights (88, 75, and 72 flights in the 100, 70, and 40 cm corridors, respectively). Bat 4 emitted an average of 32.8 calls/s in the 100 cm corridor, 36.2 calls/s 70 cm corridor, and 42.4 calls/s in the 40 cm corridor. Bat 4 flew at 3.8, 4.3, and 3.7 m/s on average in the 100, 70, and 40 cm corridors, respectively.

### Effect of corridor width on IPIs

**Figure 2:**
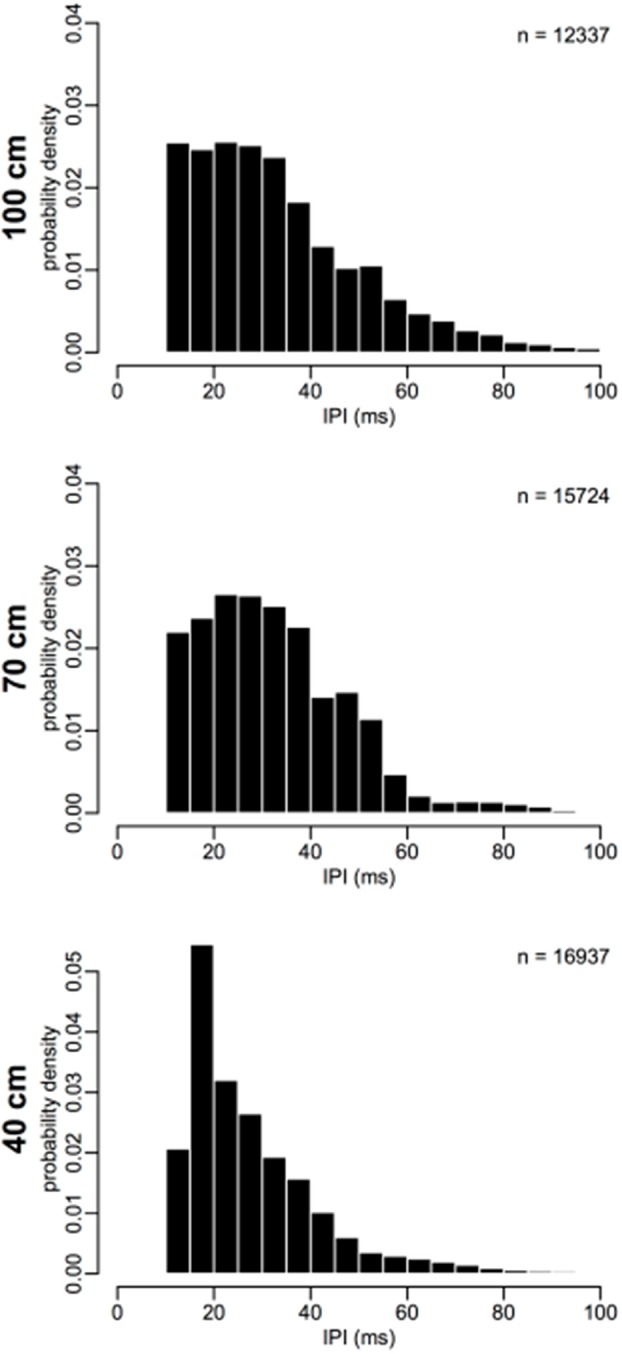
**Inter-pulse interval distributions**. IPIs from each corridor width are binned into 5 ms bins, and plotted as a probability density (total histogram area = 1). Probability of short IPIs 15–25 ms increases and probability of long IPIs 40–60 ms decreases as corridor width narrows. Number of IPIs (n) for each plot is shown as a legend in the upper-right corner. *N* = 44998.

Figure 2 shows histograms of IPIs measured from each corridor width. Table 1 reports means for call pre-IPI for each bat in each corridor condition. Some differences between IPIs measured from individual bats were found and were accounted for in the GLMM (Figure S1). No overall effect on IPI was discovered for the two blocks, so practice effects were negligible (Figure S2). This result suggests that IPIs were not impacted by practice in the flight room or seasonal effects on bats' alertness.

**Table 1:** **Mean pre-IPI for each bat in each corridor width**.

	**100 cm**	**70 cm**	**40 cm**
Bat 1	39.8	38.9	36.1
Bat 2	30.8	29.1	25.4
Bat 3	38.8	36.6	30.8
Bat 4	31.2	28.2	23.9

**Figure 3:**
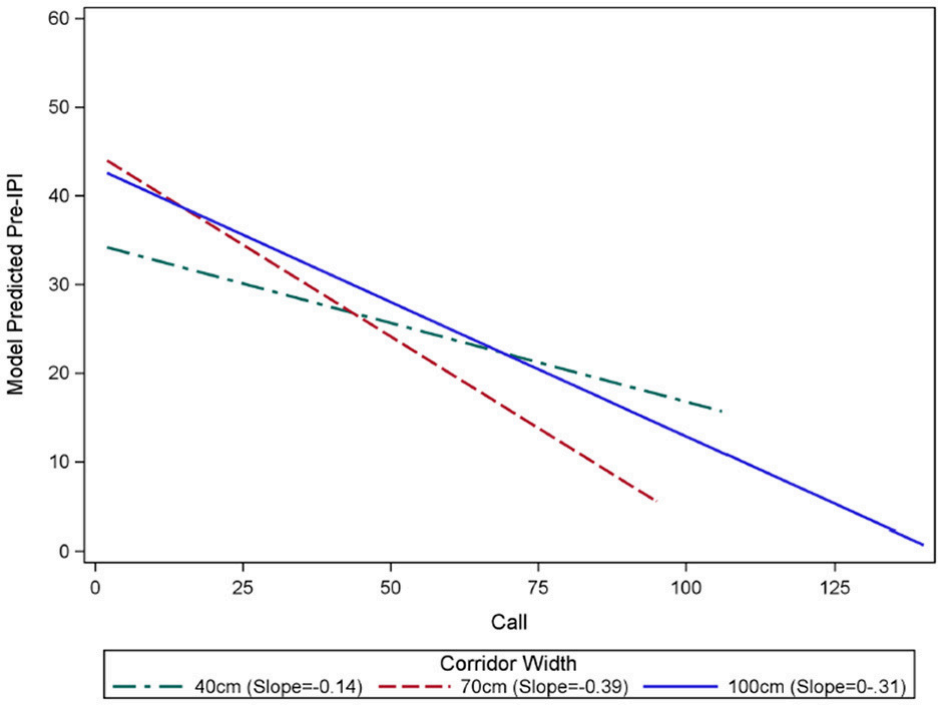
**Rates of decline in IPI over the course of flights in each corridor width**. Generalized linear mixture modeling discovered an interaction between call number within a flight “Call” and IPI. The slope of pre-IPI over successive calls within a flight was significantly different in each corridor width (green = 40 cm, red = 70 cm, blue = 100 cm). Flights in the 100 cm wide corridor had an average starting IPI of 45.06 ms, and this IPI declined by 0.31 ms with each successive call in a flight. Flights in the 70 cm-wide corridor had an average starting IPI of 44.48 ms, and this IPI declined by 0.39 ms with each successive call in a flight. There was no significant difference between the starting IPIs in the 100 cm and 70 cm corridors (*p* = 0.5395). The average starting pre IPI for flights in the 40 cm corridor was 34.24 ms, and this IPI declined by 0.14 ms with each successive call in a flight. The starting IPI for flights in the 40 cm corridor was significantly shorter than the other corridor widths (*p* < 0.0001). The slopes of the model predicted IPI over the course of flights in each corridor width were significantly different from one another (*p* < 0.0001), with the 70 cm condition showing the fastest decline, and 40 cm having the slowest decline.

The results of the GLMM are reported in Table 2. IPI was expected to decline as the bat flew closer to the back wall because the overall size of the scene decreased, and the time interval required for an echo to be received by the bat in flight consequently decreased. However, the IPI at the beginning of the flight (model-predicted pre-IPI for call 2) under the 40 cm condition (34.24 ms) was significantly shorter than both 70 cm (44.48 ms, *p* < 0.0001) and 100 cm (45.06 ms, *p* < 0.0001) conditions. There was no significant difference between IPI under the 70 and 100 cm conditions at the beginning of the flight (*p* = 0.5395). The rate of overall change in IPI (slope of the line showing progressive IPI shortening) during flights was different for each corridor width (See Figure 3). With each successive call in a flight, IPI declines significantly faster under the 70 cm condition than the 100 cm condition (*p* < 0.0001). IPI declined significantly slower under the 40 cm condition than either the 70 cm (*p* < 0.0001) or 100 cm (*p* < 0.0001) conditions.

**Table 2:** **GLMM results for pre-IPI**.

**Parameter**	**Estimate (95% CI)**	***p*-value**
Intercept	45.06 (37.61, 52.50)	
Call	−0.31 (−0.32, −0.29)	<0.0001
40 vs. 100 cm	−10.82 (−12.70, −8.93)	<0.0001
70 vs. 100 cm	−0.58 (−2.42, 1.27)	0.5395
40 vs. 70 cm	−10.24 (−11.95, −8.52)	<0.0001
Slope for call (40 vs. 100 cm)	0.17 (0.15, 0.18)	<0.0001
Slope for call (70 vs. 100 cm)	−0.08 (−0.09, −0.06)	<0.0001
Slope for call (40 vs. 70 cm)	0.25 (0.23, 0.26)	<0.0001

### Effect of corridor width on sonar sound grouping behavior

**Figure 4:**
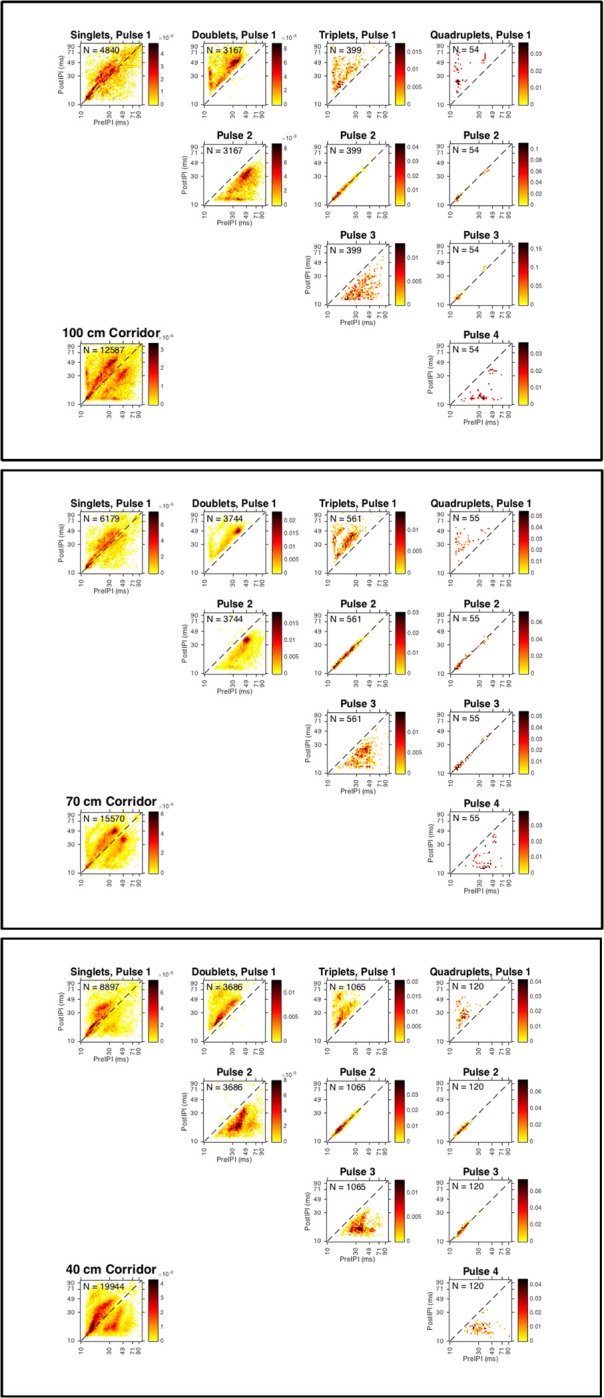
**Strobe groups for all bats in each corridor width**. Discrete probability distributions for calls emitted by all bats in the 100 cm (top panel), 70 cm (middle panel), and 40 cm (bottom panel) corridors. Heat maps show high-density (red) and low-density (yellow) pre-IPI (x-axis, all plots) vs. post-IPI (y-axis, all plots) probability. Lower left plot shows total dataset for each corridor width. Each plot is shown with logarithmic axes to emphasize the extreme ends of the distribution, and the diagonal dashed line indicates the locations where pre and post IPIs are equivalent. Number of sonar sound groups (N) having 1 (singles), 2 (doublets), 3 (triplets), and 4 (quadruplets) are reported in the upper left legend for all group types (columns). Probability distributions of each successive echolocation pulse (rows) within each group type (columns) are shown. Sound groups were assigned using the definition proposed by Kothari et al. (2014).

Calls were categorized as singles (*N* = 1), doublets (*N* = 2), triplets (*N* = 3), and quadruplets (*N* = 4) (Figure 4). Table 3 reports the percentage of calls from each experimental condition (corridor with) found in each type of sonar sound group. Using the Kothari criteria, the majority of all sounds analyzed in this experiment were classified as singles and doublets. When averaged across bats, the proportion of sounds categorized into each group subset showed no statistically significant differences between corridor widths. Singles, χ^2^_(2, **N** = 19, 916)_ = 0.0051, *p* = 0.99, doublets χ^2^_(2, **N** = 10, 597)_ = 0.023, *p* = 0.99, triplets χ^2^_(2, **N** = 2, 025)_ = 0.020, *p* = 0.99, and quadruplets χ^2^_(2, **N** = 229)_ = 0.0029, *p* = 0.99, occurred with the same statistical probabilities across corridor widths.

**Table 3:** **Percentage of calls (n) emitted in strobe groups having N sounds per group**.

**N**	**100 cm**	**70 cm**	**40 cm**
	***n* = 12,587**	***n* = 15,570**	***n* = 19,944**
1	38.5%	39.7%	44.6%
2	50.3%	48.1%	37.0%
3	9.5%	10.8%	16.0%
4	1.7%	1.4%	2.4%

As the corridor width narrowed, a greater percentage of calls were classified as triplets: 9.5% at 100 cm, 10.8% at 70 cm, and 16% at 40 cm. A similar trend was observed for quadruplets, where 1.7% and 1.4% of calls were grouped in quadruplets in the 100 and 70 cm conditions, respectively, vs. 2.4% in the 40 cm condition. A decrease in the percentage of calls classified as doublets was observed as the corridor width narrowed: 50.3% at 100 cm, 48.1% at 70 cm, and 37.0% at 40 cm. An increasing percentage of calls classified as singles was also observed: 38.5% at 100 cm, 39.7% at 70 cm, and 44.6% at 40 cm.

Figure 4 illustrates the relationship between pre- and post-pulse intervals across the entire dataset. These heat-maps show that there is a strong covariance between pre and post IPI, even for calls classified as singles. The high-density areas of the plots show the most probable pre-IPI-post-IPI pairs for the subset of call types specified in each plot. As the corridor width narrows, calls classified as within a strobe group—pulse 2 in a triplet or pulses 2 and 3 in a quadruplet—shift toward shorter IPIs (toward the lower left corner of each plot). This suggests that within strobe-group IPIs become shorter with decreasing corridor width. Figure 4 shows that this trend is particularly evident for quadruplets in the 40 cm condition.

### Effect of corridor width on IPI ratios

Since sonar sound groups are defined by the intervals preceding (pre-IPI) and following (post-IPI) each sound, the ratio of the post-IPI to pre-IPI was calculated for each call emitted in this experiment except for the last call in each flight (because the last call has no post-IPI). To investigate how the intervals before and after each call are related to one another, these ratio data were employed as a novel metric for analyzing echolocation calls and were analyzed statistically first using a generalized linear mixed model (GLMM) and second using a finite mixture model (FMM).

#### Generalized linear mixed model

**Figure 5:**
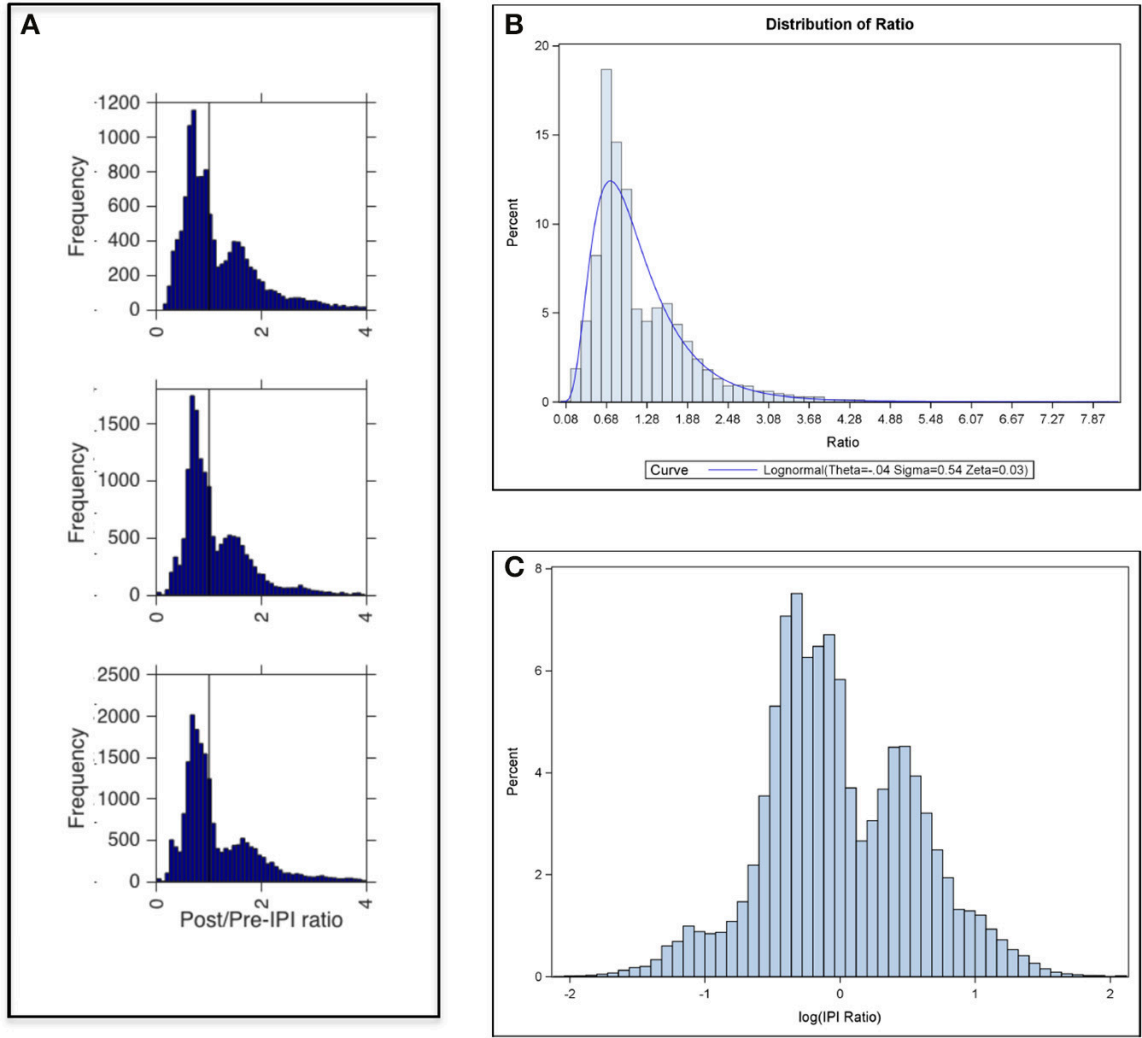
**Ratio of Post IPI to Pre IPI. (A)** Frequency of post/pre-IPI ratios in the 100 cm (top panel), 70 cm (middle panel), and 40 cm (bottom panel) corridor widths. Peaks of bimodal distributions are ratios of ~0.7 and ~1.5. Vertical line marks a ratio of 1 (where pre IPI = post IPI). **(B)** Percent distribution of post-IPI to pre-IPI ratios across all corridor widths with a lognormal curve overlay. **(C)** Same plot as in **(B)** with x-axis showing log-transformed IPI ratios revealed a bimodal distribution.

Testing for the significance of the random effects, resulting intra-class correlations (ICC) revealed that more than 99% of the variance in the ratio data is between calls, regardless of which bat, day, or flight the data is taken from (Table S2). Table 4 reports the GLMM parameter estimates. Since the slope of call is not significant (*p* = 0.4077), there are no systematic changes in IPI ratio over the course of a flight, in contrast to the absolute IPI, which declines over the course of a flight (Table 2). There is a significant difference in IPI ratio between all corridor contrasts except for 40 cm and 100 cm (*p* = 0.3524). The average IPI ratio was 1.18 for the 100 cm corridor, 1.12 for the 70 cm corridor, and 1.17 for the 40 cm corridor. However, these means do not reflect the peak IPI ratios. Figure 5A displays histograms that show the distribution of IPI ratios across all bats for each corridor width. Peak ratios are all below 1 (vertical line in each histogram from Figure 5A). Figure 5B illustrates that the underlying assumption of IPI ratio distribution normality in the GLMM is not met; rather, the distribution of IPI ratios is approximately lognormal. The log-transformed distribution of IPI ratios is shown in Figure 5C. This transformation reveals a bimodal distribution of IPI ratio. Since the statistical results of the GLMM did not take the bimodality into account, rather the GLMM averaged over the bimodal distribution to force a unimodal one, a finite mixture model was fit to investigate the effect of corridor width on the log-transformed IPI ratio distribution.

**Table 4:** **GLMM results for IPI ratio**.

**Parameter**	**Estimate (95% CI)**	***P*-value**
Intercept	1.1845 (1.1456, 1.2233)	
Call slope	0.000165 (−0.00018, 0.000509)	0.4077
40 vs. 100 cm	−0.01314 (−0.04083, 0.01455)	0.3524
70 vs. 100 cm	−0.06032 (−0.08758, −0.03306)	<0.0001
40 vs. 70 cm	0.04718 (0.02162, 0.07275)	0.0003

#### Finite mixture model

The results of the FMM are reported in Table 5. In this model, each echolocation call emitted by the bats in this experiment was assigned to one of two lognormal post-to-pre IPI ratio distributions (Figure 5C). The first distribution (higher peak, distribution 1 in Table 5) was not statistically dependent on corridor width; however, the second distribution (lower peak, distribution 2 in Table 5) was statistically dependent on corridor width. With a larger corridor width, the log(IPI ratio) significantly increased. These distributions hold true when adjusted for bat, day, and flight (Table S3), but they do not fully account for the hierarchical correlation structure of the data, as did the generalized linear mixture model. The results of the FMM show that when the bimodal distribution, (treated as a unimodal distribution in the GLMM), is instead treated as two separate distributions, the decreasing corridor width significantly decreases the IPI ratio of the second distribution, whereas the first distribution of IPI ratios is not significantly affected by corridor width. These results suggest that IPI ratios resulting from calls within a group may decrease as the corridor width decreases. This would produce shorter overall IPIs, and also progressively smaller differences between post-IPI and pre-IPI. The results of the FMM also might explain why the GLMM did not show a significant difference between IPI ratios from the 40 vs. 100 cm contrast.

**Table 5:** **FMM bivariate lognormal IPI ratio results**.

**Distribution**	**Parameter**	**Estimate (95% CI)**	***P*-value**
1	Intercept	0.07593 (0.06308, 0.08879)	
1	40 vs. 100 cm	−0.01071 (−0.02716, 0.005748)	0.2022
1	70 vs. 100 cm	−0.00722 (−0.02396, 0.009519)	0.3978
1	40 vs. 70 cm	−0.003486 (−0.01899, 0.01202)	0.6594
2	Intercept	−0.3582 (−0.3721, −0.3444)	
2	40 vs. 100 cm	0.08565 (0.06879, 0.1025)	<0.0001
2	70 vs. 100 cm	0.06181 (0.04565, 0.07796)	<0.0001
2	40 vs. 70 cm	0.02384 (0.01014, 0.03754)	0.0006

### Comparison of IPI and IPI ratio results

**Figure 6:**
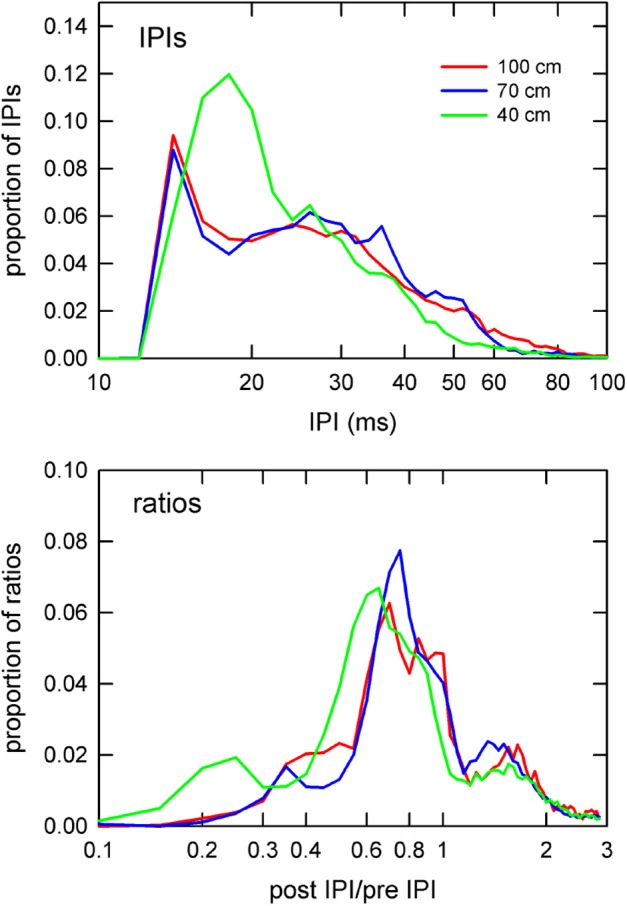
**Distributions of IPIs and IPI ratios**. Proportion of IPIs (top panel) and post IPI/pre IPI ratios (bottom panel) by corridor width. IPIs and ratios are plotted on logarithmic x-axes to emphasize differences at the extreme low end of the IPI and ratio ranges. The 40 cm curve (green) shows drastically higher proportions of sounds having 20 ms IPIs (top panel) than in the other corridor widths. Similarly, the 40 cm curve for the ratios shows a shift toward smaller ratio values in comparison to the other widths.

Figure 6 highlights the main differences between corridor widths according to absolute IPI and post-/pre-IPI ratio. The largest difference between corridor widths for IPI was in the short-IPI range around 20 ms. The 40 cm corridor width showed a drastically higher proportion (12%) of 20 ms long IPIs compared to the 70 and 100 cm corridor width conditions (5%). Furthermore, the proportion of IPIs in the 30–60 ms range was lower in the 40 cm width compared to the other widths (Figure 6, top panel). The IPI ratios offer a more comprehensive, albeit complex result. Post-IPI to pre-IPI ratios of 0.1–0.3 were more prevalent in the 40 cm condition than the other widths. Additionally, the greatest proportion of ratios in the 40 cm condition was 0.6, as compared to the other widths, which had ratio peaks of 0.7 (100 cm) and 0.8 (70 cm). Ratios between 0.9 and 1.5 were less prevalent in the 40 cm condition than the other two corridor widths. The 40 cm condition appears drastically different from the 70 and 100 cm conditions in both parts of Figure 6, demonstrating a shift toward both shorter IPIs and smaller ratios.

## Discussion

In this study, the relationship between successive IPIs during in-flight bat echolocation in cluttered surroundings was investigated. The task required sustained steering and obstacle avoidance behavior over several meters of flight in dense clutter. With decreasing corridor width, IPIs became shorter, and the proportion of sonar sound groups having 3 or more calls increased. The results of this study support the idea that bats use pulse groups to create a stable auditory image in complex auditory scenes (Surlykke and Moss, 2000; Melcón et al., 2011; Moss et al., 2011; Falk et al., 2014; Kothari et al., 2014; Sändig et al., 2014). They also clearly implicate the immediate proximity of obstacles as a particularly critical factor in the bat's adaptive vocal behavior (Petrites et al., 2009; Knowles et al., 2015). The evidence presented here also suggests that precise timing of echolocation call emission is important for simultaneous long-range and short-range auditory imaging, and that pulse-echo ambiguity can be controlled using this strategy.

### Sonar sound groups are advantageous for clutter navigation

In this experiment, as clutter proximity became closer, the bats shortened their mean IPI (Table 1) and starting IPI (Figure 3). The proportion of short IPIs increased as the corridor width was narrowed (Figures 2, 6). A previous study used similar methods to the current study to show that *E. fuscus* emitted shorter IPI calls as periodic clutter became denser (Petrites et al., 2009), and that strobe groups dominated the stream of echolocation pulses at a corridor width of 100 cm—the minimum width used in this study. The present experiment used nearly identical chain spacing as the previous one, but began with a corridor width of 100 cm as a starting point. Based on the results of the current study and previously published evidence (Petrites et al., 2009; Sändig et al., 2014), inter-pulse interval patterning is likely advantageous during obstacle avoidance tasks. One widely used obstacle-avoidance paradigm measures changes in pulse parameters as bats maneuver to avoid thin wire obstacles (Hahn, 1908; Griffin and Galambos, 1941; Sändig et al., 2014). Sändig et al. (2014) found that a more difficult wire-avoidance task (e.g., when wires were spaced closely together) yielded shorter-IPI calls and increased the number of sonar sounds per group. In that experiment, the ability of the bats to precisely locate each stretched wire determined their success; and the bats that used more sounds per strobe group when approaching the wire obstacles were more successful in navigating between them. Since performance data was not used in the present study (because very few failed flights occurred), we were unable to determine whether the emission of more sounds improved performance. However, the significant decrease in IPI length in the closest-proximity clutter condition (40 cm) compared to the other chain configurations, suggests that an increased pulse repetition rate in the 40 cm configuration was instrumental for successful navigation. Additionally, the increase in percentage of triplets and quadruplets as corridor width narrowed (Figure 4, Table 3) support the conclusions drawn by Sändig et al. (2014). The results of the current study support the previous suggestion that the emission of sound groups in this environment increases navigational success (Petrites et al., 2009; Falk et al., 2014).

### IPIs are influenced by the occurrence of ambiguous echoes

There are two possible solutions to pulse-echo ambiguity in clutter navigation. The first is shifting the frequencies of successive pulses so their echoes can be processed separately. A frequency-hopping strategy is used by several kinds of echolocating bats (Kalko, 1995; Mora et al., 2004; Guillén-Servent and Ibáñez, 2007; Jung et al., 2007). Big brown bats can shift frequencies to avoid jamming in open environments (Bates et al., 2008; Chiu et al., 2009; Moss et al., 2011); however, to locate targets while resisting interference from clutter, big brown bats rely on spectral and temporal comparisons between the emitted signal and received echoes distributed across the a wide frequency band (Kalko and Schnitzler, 1998; Moss et al., 2006; Hiryu et al., 2010; Bates et al., 2011; Simmons, 2012, 2014). The use of wide-band sounds to achieve high-resolution imaging precludes pure frequency tagging because subsequent sounds occupy almost completely overlapping frequency bands. However, previous studies have shown that big brown bats do change the starting or ending frequency of neighboring calls with overlapping echo streams; meaning that slight spectral differences between two broadcasts with short IPIs may be useful in overcoming pulse-echo ambiguity (Hiryu et al., 2010; Warnecke et al., 2015). The changes in frequency found in one study were only about six percent of the overall signal bandwidth, but this may be enough to disambiguate pulse-echo pairs (Hiryu et al., 2010). Although these signals are very highly correlated (i.e., completely ambiguous to a matched filter receiver), there may be additional cues in the precise time-frequency structure that are useful for bats to disambiguate individual echoes or echo packets (Hiryu et al., 2010; DiCecco et al., 2013).

**Figure 7:**
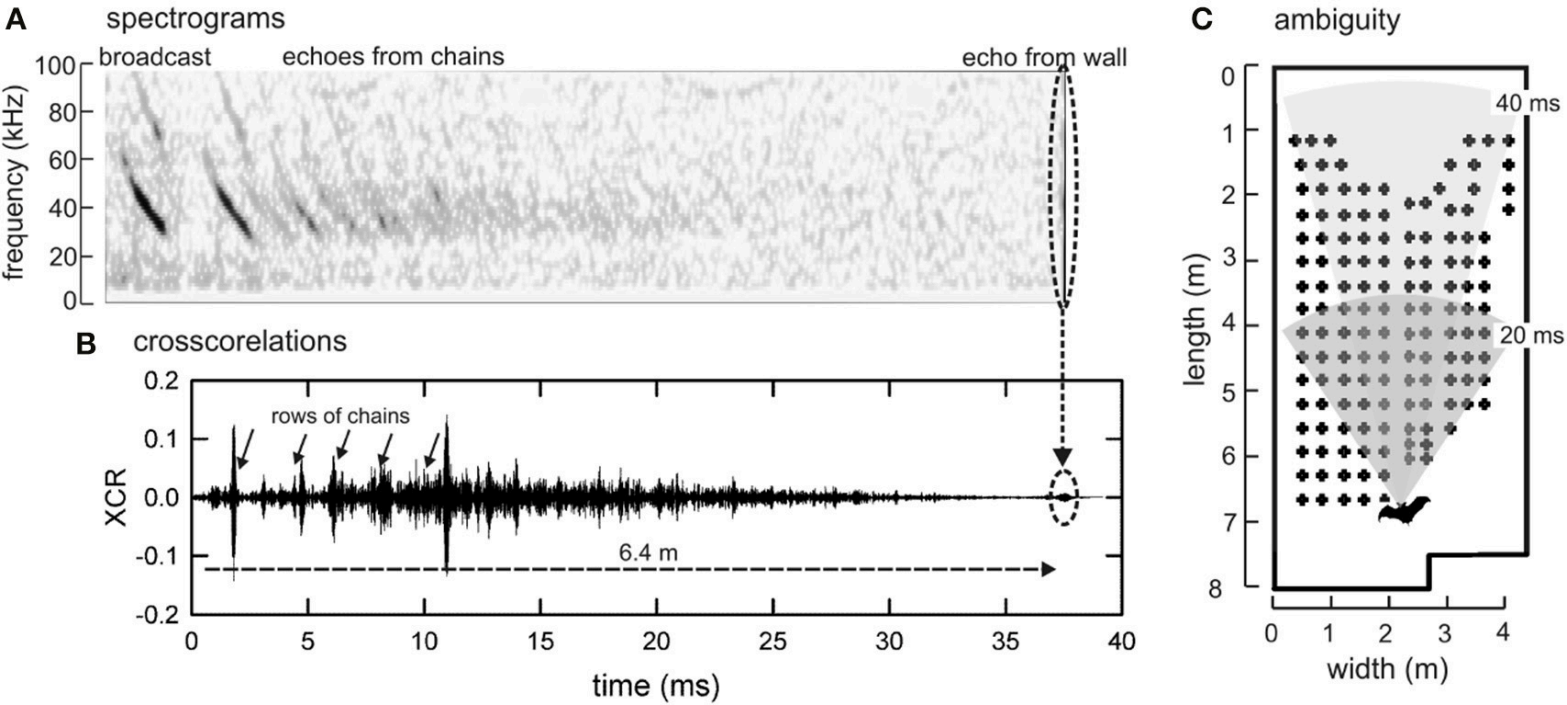
**Pulse-echo ambiguity. (A)** Spectrograms of one FM echolocation broadcast and the series of echoes generated during flight in the 40-cm corridor by this broadcast from successive rows of chains and then from the end wall. These echoes were recorded by a microphone placed behind the bat early in its flight (when the bat was 6.4 m from the end wall) and aimed to point along the corridor (technique from Hiryu et al., 2010). **(B)** Cross correlation function between the broadcast and the entire stream of echoes used to compress FM sweeps as a display to maximize visualization of rows of chains. Note epoch of echo reception lasting about 37 ms, corresponding to distance to wall. **(C)** Plan view of room with superimposed arc sectors depicting distance broadcast travels to reach location where echoes return after 20 ms (dark gray sector) or 40 (light gray sector). If IPIs are shorter than 40 ms, echoes of the first sound in a pair arrive after the second sound is emitted, creating conditions for pulse-echo ambiguity. Longer-delay echoes of the first sound can be mistaken for short-delay echoes of the second sound.

Figure 7 illustrates how pulse-echo ambiguity occurs in this experiment. Perception of phantom objects at short range can occur, and alternating short and long IPIs can tease out real objects from false ones. The emission of doublets lets the bat probe the entire scene with long IPIs and then probe quickly for objects in the nearer part of the scene that require more immediate reactions with short IPIs (Simmons et al., 1998; Stimson, 1998; Melcón et al., 2011). When a doublet is emitted, ambiguous echoes fail to appear on alternating pulses, which separate phantom objects from the real objects, which appear on every pulse and solidify as components of a unified perceptual scene. This is important because multiple echoes at the same delay are required for the neural representation of an object to solidify as true objects (Surlykke, 2004).

The present results show that a decrease in IPI accompanied the increase in call number per group (Figure 4), which suggests that in order to successfully navigate the chain array down the 40 cm corridor, bats risk potential pulse-echo ambiguity caused by IPIs of around 20 ms in order to achieve the spatial resolution necessary for avoiding collisions with chain obstacles. The threat of ambiguity may also have influenced the bats' rate of IPI decline observed over the course of each flight (Figure 3). The slower rate of IPI shortening observed for the 40 cm condition is likely due to the lower starting IPI, and the balancing act between reducing ambiguous echoes and avoiding close-proximity chains. At the end of the flights in the 40 cm condition, the GLMM predicted longer IPIs based on this slope; however, in practice, all bats emitted a landing buzz for which some IPIs were too short (< 12 ms) to be included.

The bats in this experiment emitted echolocation calls primarily at oscillating time intervals (doublets). Heat-map clusters in Figure 4 suggest that as the pre-IPI becomes longer or shorter, the post-IPI increase or decreases in proportion. This precise distribution of probable IPIs before and after each sound likely exploits temporal feature detectors in the bat's auditory cortex such as delay-tuned neurons (Dear et al., 1993), which are ultimately responsible for producing a cohesive auditory scene free of ambiguous echoes. The precise timing of echolocation calls over repeated flights suggests that a motor pattern generator could be involved in sonar sound group production.

### Other potential uses for sonar sound groups

Sonar sound groups may also be used as an adaptive mechanism for discriminating an insect prey target from background clutter, or tracking target motion. The current study did not use an insect target, but previous work has shown that the target's location amidst clutter impacts echolocation IPI patterning. For example when a prey target is located in close proximity to background clutter, big brown bats emit more sonar sound groups than when tracking a target in an open room (Moss and Surlykke, 2001; Moss et al., 2006; Kothari et al., 2014). Moss et al. (2006) found that *E. fuscus* emits a greater proportion of sonar sound groups with decreasing distance between the prey target and background clutter. Kloepper et al. (2014) showed that, while tracking a moving object, *E. fuscus* emitted doublets with IPIs in a bimodal distribution with 45 ms as the dividing line between short (< 45 ms) and long (>45 ms) IPIs. Similarly, bats tracking a moving target with an unpredictable trajectory emitted 90% of their sounds in doublets with a mean IPI of 44.7 ms, and emitted a greater proportion of sounds in groups than when the moving target had a predictable trajectory (Kothari et al., 2014). This is consistent with results from another study that showed a 70–90% incidence of doublets, but an increase in the incidence of triplets with greater task difficulty (Kothari et al., 2014). The flight path trajectory may also influence sound patterning. When attacking prey, big brown bats emit sounds with a different temporal patterning depending on whether the bat approaches the prey target directly or indirectly (Moss and Surlykke, 2001; Moss et al., 2006). In general, it seems that big brown bats emit more sonar sound groups as the difficulty of a task increases (Moss and Surlykke, 2001; Moss et al., 2006; Falk et al., 2014; Kothari et al., 2014; Fawcett et al., 2015).

One explanation for the strict temporal patterning of echolocation pulses observed in this study is that pulse emissions could be coupled to the wing-beat and inferred respiration cycle (Moss et al., 2006; Koblitz et al., 2010). This possibility was not directly investigated here, however, coupling sonar sound groups to respiration and flight movements is a probable energetic benefit in some situations. Alternatively, a strict dependence of IPI on wing beat cycle could be disadvantageous for bats flying in cluttered environments where sophisticated aerial maneuvering may require de-coupling of wing motion and sound production (Moss et al., 2006; Kothari et al., 2014). While it is logical that the long post-IPIs associated with the last call in a sound group, or the pre-IPI of the first sound in a group are limited in duration by the minimum time required to inhale, a strict dependence of pulse intervals made during exhalation on the wing beat cycle is improbable. The reason for this is that evidence presented here and elsewhere suggests that sonar sound grouping behavior serves an important function for bats avoiding obstacles or discriminating insect prey from close-proximity clutter. While we concede that inhalation is a limiting factor in sonar sound group patterning, we argue that the *primary* purpose for structuring sonar sounds into groups is perceptual and not energetic.

### Future directions

#### Does spatial memory influence IPI patterning?

Perception of the auditory scene, like most other modes of perception, may also be influenced by expectation or memory (Surlykke, 2004; Moss et al., 2006; Nijhawan, 2008; Barchi et al., 2013; Kothari et al., 2014). In the present study, no effect of practice was found (Figure S2), which shows that improved memory over repeated flights did not change IPI length or ratio. This study did not explicitly investigate the influence of spatial memory or experience on echolocation call timing. While there is evidence that spatial memory is important for optimizing flight paths (Barchi et al., 2013; Knowles et al., 2015), the current results suggest that observed strobe groups and short IPIs are purely the product of the sensory demands of the environment (Moss et al., 2006; Kothari et al., 2014). However, this question warrants further investigation. In the current study, the flight path was completely straight; future work might consider the benefits and costs of sonar sound grouping in a more complex flight path.

#### Future directions in IPI ratio modeling: moving beyond sonar sound groups

In this study, sonar sound groups were classified using the criteria put forth by Kothari et al. (2014). While this set of criteria was instrumental in quantitatively defining sonar sound groups, the Island Criterion, whereby groups of sounds are defined as having a between-group IPI of at least 1.2 times the within-group IPI, may need to be decreased in order to accommodate sounds emitted in extreme clutter, such as the 40 cm condition used in this experiment. The reason being that it is possible that some sounds in this condition could have been inappropriately categorized as singles. In Table 3, the sounds emitted by bats flying in the 40 cm condition showed an increase in singles, triplets, and quadruplets, but a decrease in percentage of doublets. This could be due to limitations imposed by Island Criterion, and may not accurately describe the behavior in this extremely cluttered environment. Utilizing the IPI ratio as a metric may offer an alternate quantitative measure for investigating temporal patterning of echolocation calls without the restrictions necessary to define sound groups. Instead of identifying individual sonar sound groups and analyzing within and between group intervals, a future approach might evaluate the entire stream of echolocation sounds to elucidate the global temporal pattern used by echolocating big brown bats, and move away from painstaking statistical analyses of sonar sound groups. Such a change in analyses among the biosonar research community might allow for greater ease of comparison between independent experiments, and would allow for overarching questions about whether bats emit fundamentally similar or different sound patterns in different behavioral contexts, to be answered more easily.

Perhaps the most intriguing result of this experiment was that the bivariate lognormal distributions of IPI ratio had one component distribution that was sensitive to corridor width and another that was not (Figure 5, Table 5). This suggests that calls having particular IPI ratios are the result of a clutter-dependent behavior—Distribution 2 in Table 5—whereas calls having IPI ratios belonging to Distribution 1 are the result of clutter-independent echolocation behavior. These results should be interpreted with caution because the FMM fit here explicitly assumed that the log-transformed ratio values were distributed according to two normal distributions. The distributions may, in fact, be more complex. Finite mixture models offer a promising analytic framework for this type of data. It seems likely that multiple unobserved mechanisms impact bat IPI, and FMM allows inferences to be made about those mechanisms. Here, proximity of the chain obstacles impacted one of two IPI ratio distributions. It would be interesting to test whether this holds true for other experimental paradigms.

In conclusion, the results of this experiment support the idea that bats use strobe groups to achieve detailed spatial resolution in cluttered or challenging environments (Surlykke and Moss, 2000; Moss et al., 2006; Chiu et al., 2009). The strict organization of sequential IPIs seen in this experiment is interpreted as a strategy for simultaneously organizing echo streams for both proximal and distal portions of the auditory scene, thus aiding in path planning and preventing ambiguous echoes from arising (Figure 7). This strategy, in combination with spectral pulse-echo comparisons, can account for the bats remarkable ability to navigate, orient, and hunt in cluttered forest environments.

## Author contributions

AW conducted the research and wrote the manuscript, KF conducted the research and analyzed data, JG analyzed data and edited the manuscript, RS performed statistical analyses, IM conducted the research, and JS designed the experiment and wrote the manuscript.

### Conflict of interest statement

The authors declare that the research was conducted in the absence of any commercial or financial relationships that could be construed as a potential conflict of interest.
