# Supplementary material for: Echolocating Big Brown Bats, Eptesicus fuscus, Modulate Pulse Intervals to Overcome Range Ambiguity in Cluttered Surroundings
Source: Front Behav Neurosci. 2016 Jun 22;10:125. doi: 10.3389/fnbeh.2016.00125 (PMC4916216; doi:10.3389/fnbeh.2016.00125)
Supplement: Supplementary file 1 [file Presentation1.PPTX]

## Slide 1
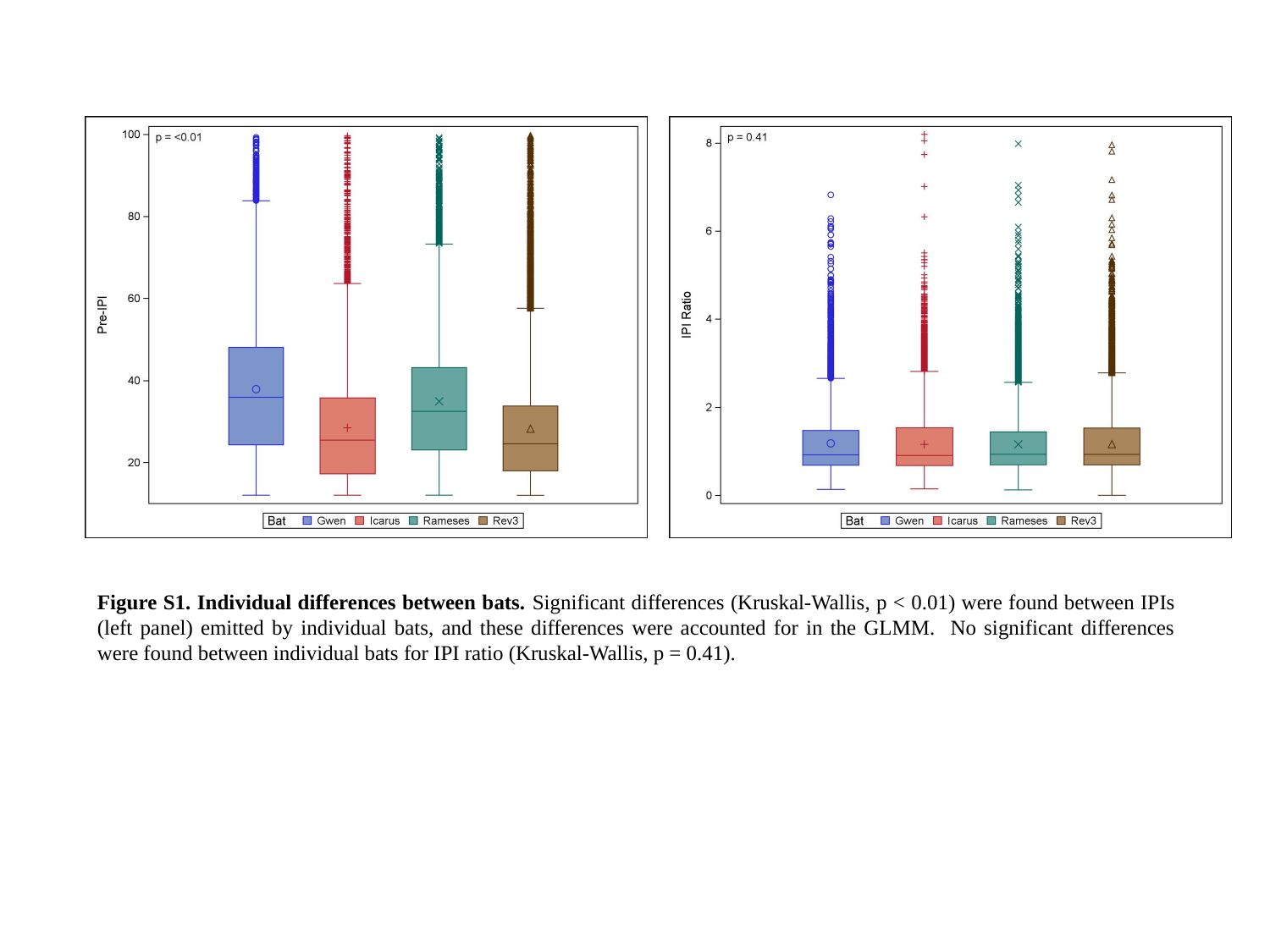

Figure S1. Individual differences between bats. Significant differences (Kruskal-Wallis, p < 0.01) were found between IPIs (left panel) emitted by individual bats, and these differences were accounted for in the GLMM. No significant differences were found between individual bats for IPI ratio (Kruskal-Wallis, p = 0.41).

## Slide 2
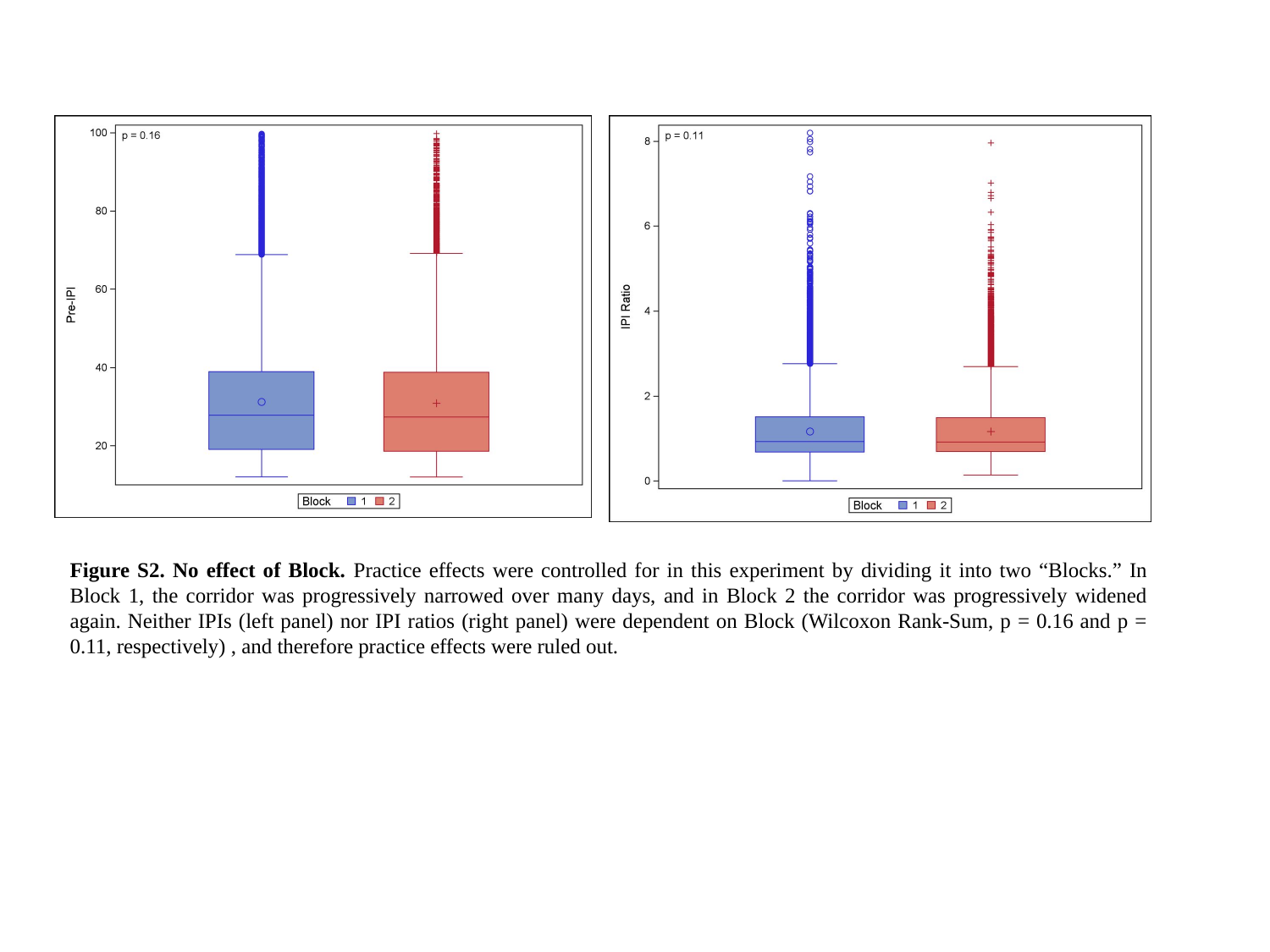

Figure S2. No effect of Block. Practice effects were controlled for in this experiment by dividing it into two “Blocks.” In Block 1, the corridor was progressively narrowed over many days, and in Block 2 the corridor was progressively widened again. Neither IPIs (left panel) nor IPI ratios (right panel) were dependent on Block (Wilcoxon Rank-Sum, p = 0.16 and p = 0.11, respectively) , and therefore practice effects were ruled out.

## Slide 3
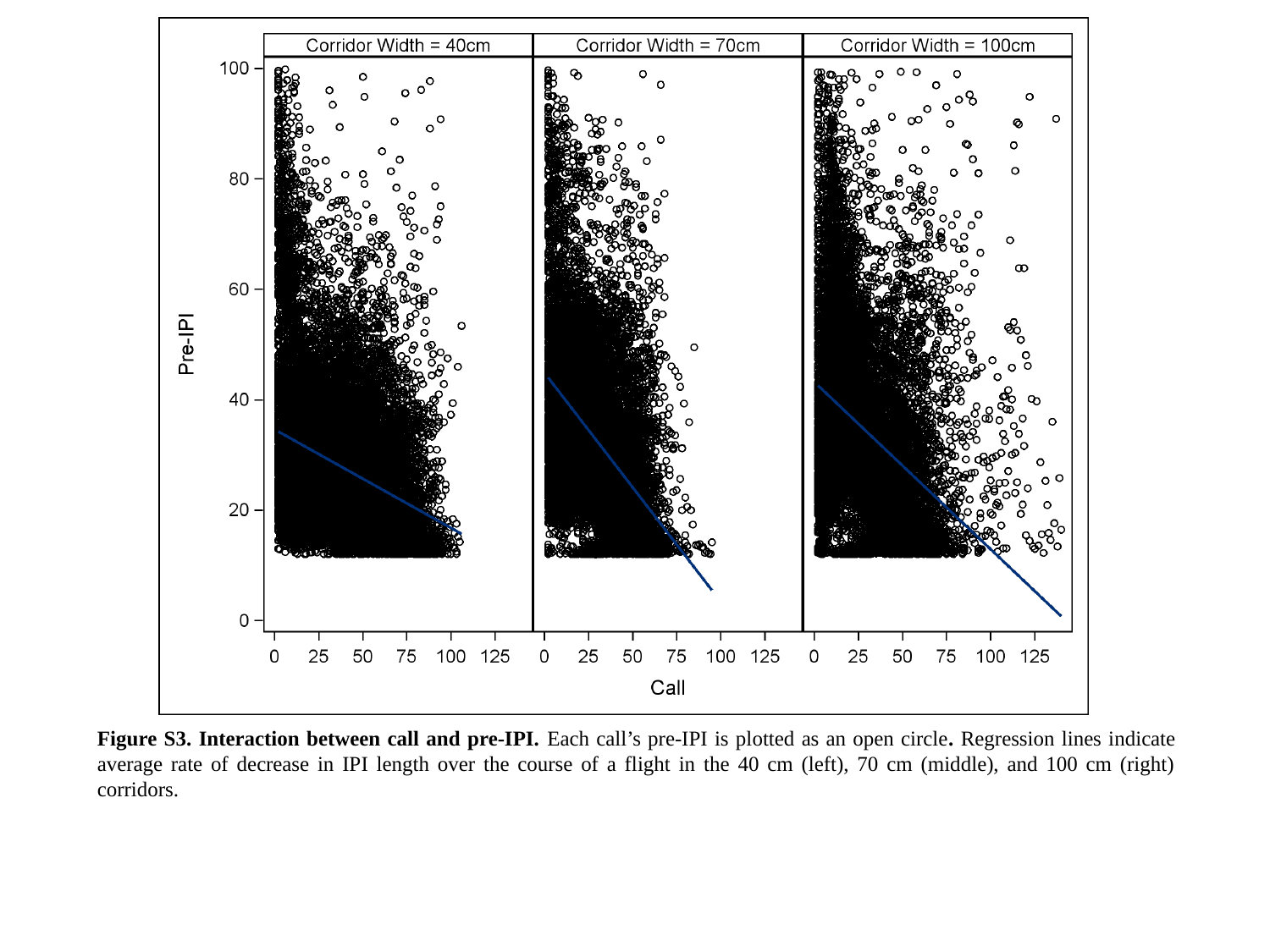

Figure S3. Interaction between call and pre-IPI. Each call’s pre-IPI is plotted as an open circle. Regression lines indicate average rate of decrease in IPI length over the course of a flight in the 40 cm (left), 70 cm (middle), and 100 cm (right) corridors.

## Slide 4
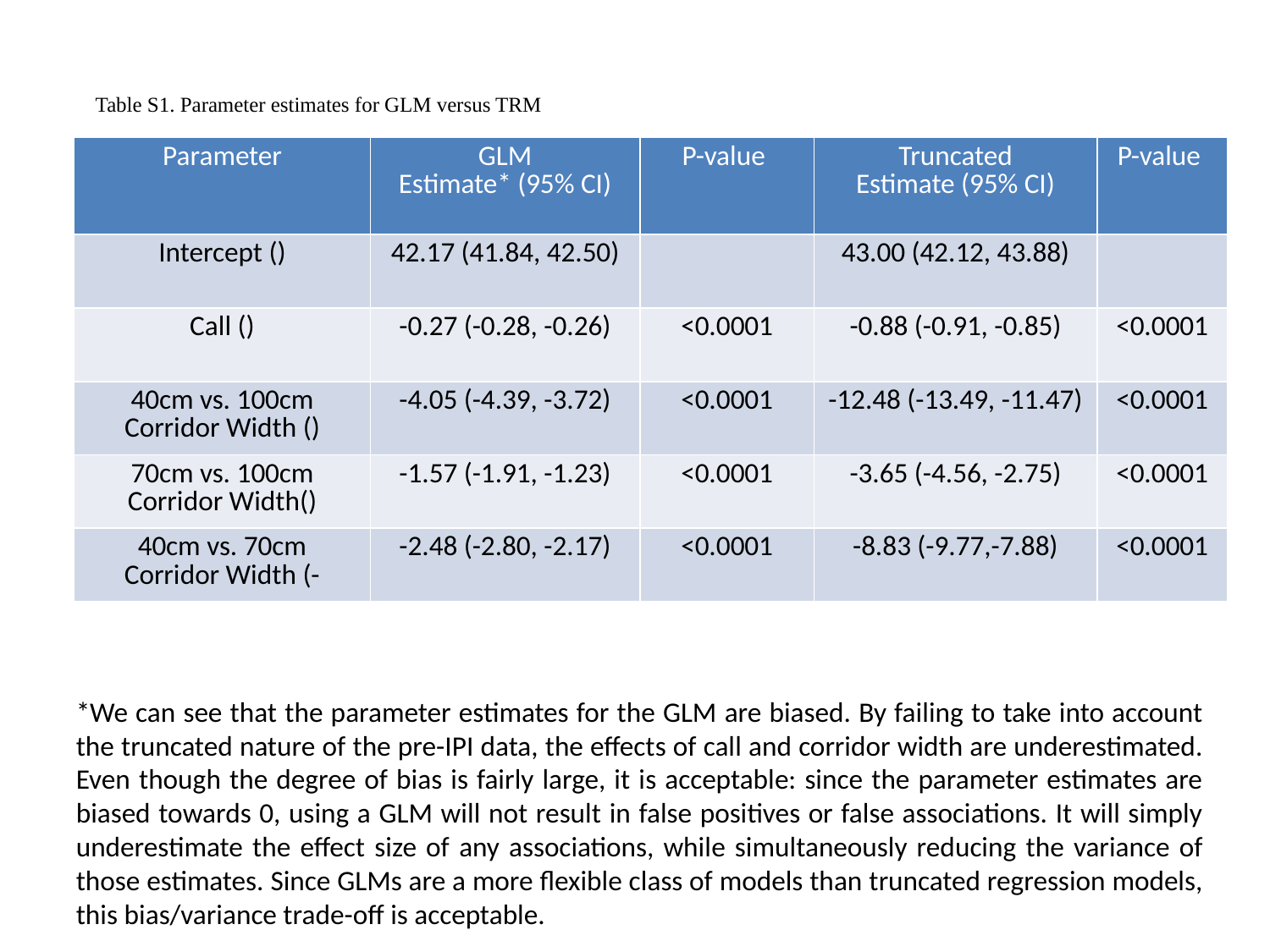

Table S1. Parameter estimates for GLM versus TRM
| Parameter | GLM Estimate\* (95% CI) | P-value | Truncated Estimate (95% CI) | P-value |
| --- | --- | --- | --- | --- |
| Intercept () | 42.17 (41.84, 42.50) | | 43.00 (42.12, 43.88) | |
| Call () | -0.27 (-0.28, -0.26) | <0.0001 | -0.88 (-0.91, -0.85) | <0.0001 |
| 40cm vs. 100cm Corridor Width () | -4.05 (-4.39, -3.72) | <0.0001 | -12.48 (-13.49, -11.47) | <0.0001 |
| 70cm vs. 100cm Corridor Width() | -1.57 (-1.91, -1.23) | <0.0001 | -3.65 (-4.56, -2.75) | <0.0001 |
| 40cm vs. 70cm Corridor Width (- | -2.48 (-2.80, -2.17) | <0.0001 | -8.83 (-9.77,-7.88) | <0.0001 |
*We can see that the parameter estimates for the GLM are biased. By failing to take into account the truncated nature of the pre-IPI data, the effects of call and corridor width are underestimated. Even though the degree of bias is fairly large, it is acceptable: since the parameter estimates are biased towards 0, using a GLM will not result in false positives or false associations. It will simply underestimate the effect size of any associations, while simultaneously reducing the variance of those estimates. Since GLMs are a more flexible class of models than truncated regression models, this bias/variance trade-off is acceptable.

## Slide 5
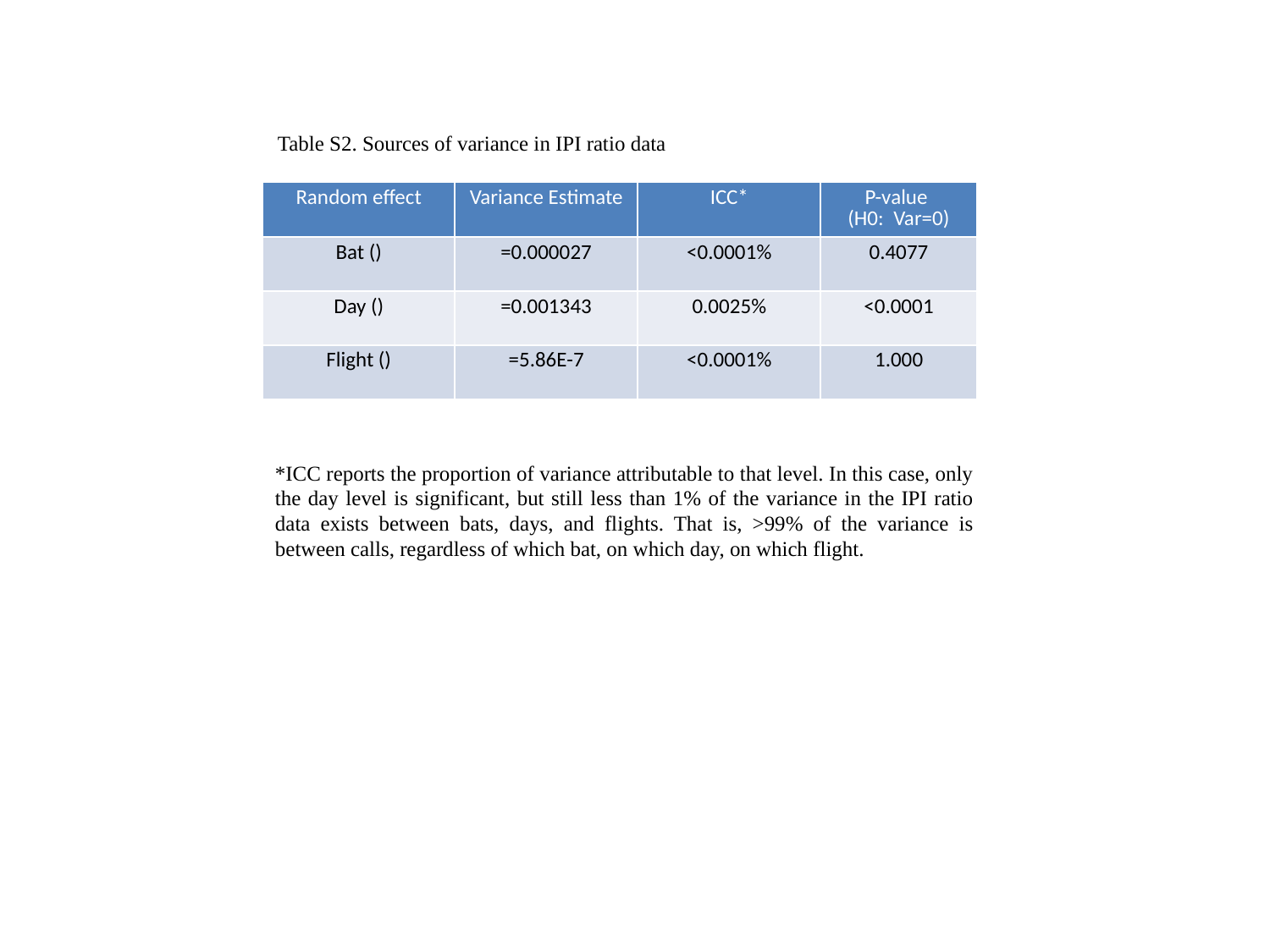

Table S2. Sources of variance in IPI ratio data
| Random effect | Variance Estimate | ICC\* | P-value (H0: Var=0) |
| --- | --- | --- | --- |
| Bat () | =0.000027 | <0.0001% | 0.4077 |
| Day () | =0.001343 | 0.0025% | <0.0001 |
| Flight () | =5.86E-7 | <0.0001% | 1.000 |
*ICC reports the proportion of variance attributable to that level. In this case, only the day level is significant, but still less than 1% of the variance in the IPI ratio data exists between bats, days, and flights. That is, >99% of the variance is between calls, regardless of which bat, on which day, on which flight.

## Slide 6
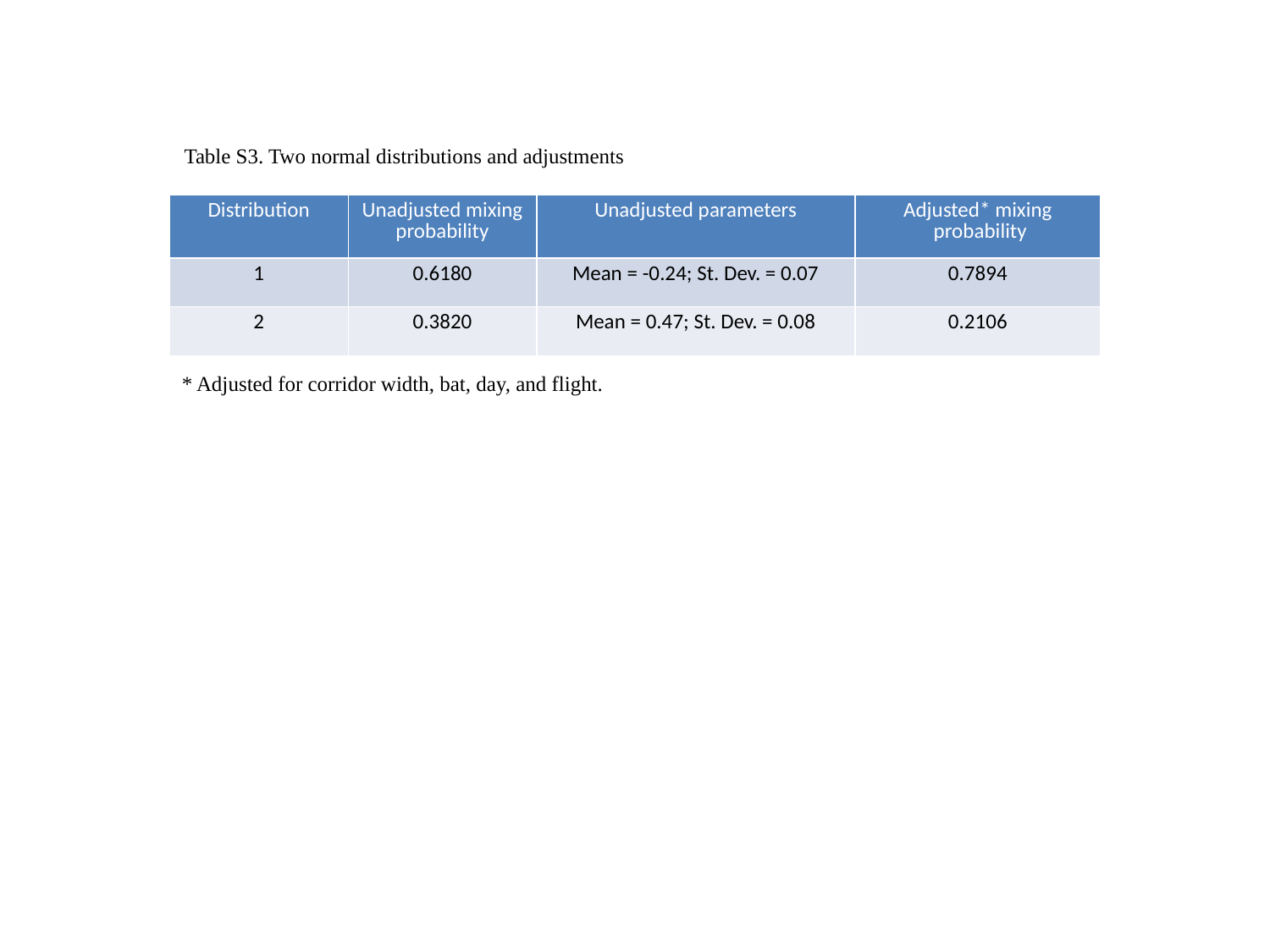

Table S3. Two normal distributions and adjustments
| Distribution | Unadjusted mixing probability | Unadjusted parameters | Adjusted\* mixing probability |
| --- | --- | --- | --- |
| 1 | 0.6180 | Mean = -0.24; St. Dev. = 0.07 | 0.7894 |
| 2 | 0.3820 | Mean = 0.47; St. Dev. = 0.08 | 0.2106 |
* Adjusted for corridor width, bat, day, and flight.
